# Physiology of the Dental Structures

**Published:** 1873-10

**Authors:** S. P. Cutler


### ARTICLE II.

### *Physiology of the Dental Structures.*—Concluded.

BY S. P. CUTLER M. D., D. D. S.

Although the hepatic functions cannot be said to be directly vital, still they have great influence on all vital acts; unlike the brain, heart and lungs, the great vital tri-pod, the hepatic functions may be suspended almost entirely for a time, without necessarily proving fatal; not so with either of the three vital centres, the functions of which cannot be entirely suspended for any length of time without fatal consequences. There are cases where the functions of one of these three organs may continue after the normal action of one or both of the others are suspended: as an instance, the heart may continue to beat after respiration has been cut off mechanically or otherwise, as in cases of hanging, drowning, or mechanical obstructions in the wind pipe, by swelling, or foreign bodies, and life recovered again, after it had apparently gone out; there are instances of suspended animation or apparent death. Although the vital functions of the heart may be said to be mechanical chiefly, still this mechanical function can not be suspended for a single minute without fatal results. Respiration, in *modus operandi*, may be said to be mechanical, although other important attributes are embraced in the respiratory processes, as the taking in of oxygen, and giving out carbonic acid and watery vapor, by both mechanical and asmatic action.

In some of the lower order of animals, æration takes place without any respiratory act; not having genuine lungs, still lung functions are carried on as in higher animals. In some others there is neither true circulation, respiration, or brain function, there being no true organs of either, still organic or vegetative life is carried on.

Man, in addition to all other functions common to all the lower animals, has the additicnal cerebral functions of a higher intelligence, space not permitting any detail. In a physiological point of view, mind may be regarded as functional, though altogether different from all other functions.

The three great vital centres all have their characteristic functions, when taken collectively; ramify so as to embrace all other collateral functions, not regarded as vital, *per se*, only relatively so; as the three great vital centres embrace all others. Whether we regard the mind as a function of the great nervous centre, the brain, or not, we can only discuss it as such in a physiological point of view. If we regard it as the result of evolution and growth, as any other function, we can readily comprehend the pathological condition when the brain is diseased, either curatively or hopelessly so, but if we view the mind as ultra physical, or an independent entity, then we have no scientific stand point of discussion. The brain has other and important functions besides mind to supervise; coming to the senses, we must look upon all knowledge as sensual, as there are no other channels through or by which any kind of knowledge can be derived; hence, knowledge is a thing of education, and governed greatly by surrounding circumstances.

If mind or knowledge be regarded (on the other hand) as super sensual and inate, then we know but little about it. All the impressions through the senses may be looked upon as so many greater and smaller waves or rythms; such being now demonstrated as the order of nature. All the dynamics of the unions consists in dynamical waves, of greater or lesser extent, and physiology forming no exception to the rule.

Let us turn our attention for a short time to the circulation of the blood; the circulating mass is confined within a system of circulatory vessels, the heart being the centre, there being no stopping point of rest anywhere in the entire rounds, because to stop, to pause is to die; hence, this function is a perpetual motion, never beginning, nor never ending, no terminal or halting point, as in case of respiration. So far as the physical aspect is concerned, the movement of the blood is mechanical, or its mechanical function. The heart, arteries, capillaries and veins, are mainly muscular or fibrous throughout, and by muscular contractility mainly, the function of circulation depends. Let us look into the circulation for a few moments: first, the heart's contraction upon the filled ventricles, supposing the vessels all full; the heart contracts and diminishes in size, and empties its chambers, forcing the blood into the arteries with such a force, as to considerably dilate them, at the same time the blood is driven forwards into and through the capillaries, into the veins, and through them back to the auricles of the heart, at each systale, facilitated by the capillaries and arteries. As the heart contracts, there is no general lessening in the mean dimensions of the system, of the circulation; as the heart lessens, the arteries expand in due proportion; as the heart opens or dilates, the vessels contract in turn to the amount of the heart's expansion; so that this constant ebb and flow is just self-compensating, and constitutes one complete circulatory wave.

The experiments made by Volkman with his hæmadrometer, on the velocity of the circulation, showed it to be about 300 millimetres a second, or nearly 12 inches; the velocity near the heart is greater than more remote, and much slower through the capillaries than arteries. A thorough knowledge of the circulation is of incalculable value to the practitioner of medicine, more especially in all acute diseases. When the finger is placed on the radial artery, certain pulsations are felt, an ebbing or flowing, a swelling and diminution in calibre felt, as though some foreign body was passing under the finger. The length, size and density of each pulse, is the chief monitor in diagnosing acute diseases; it speaks a silent but intelligent language. It is the education of the finger ends that interprets the secrets of disease known only to science. In heart diseases, much knowledge is gained by osculation, by training the ear.

Next in order of vital functions, comes the physics of respiration. Let us notice the tidal ebb and flow of the chest during inspiration and expiration, the expanding and contracting of the chest some eighteen more or less times in a minute, like the number of pulse, varying in different individuals and at different ages in the same person, in health and disease. In the act of inhalation the chest is raised upwards and expanded by the action of the inter-costal muscles, and the diaphragm straitening or flattening out, pushing out the abdomen. Ordinarily in this act, the chest is increased, and takes in from fifteen to thirty cubic inches of air, which may be voluntarily increased many times this amount. The respiratory act is known as the respiratory wave.

There is a much larger amount of residual air contained in 600,000,000 of air vesicles, that can never be forced out by any effort of the individual; the amount so residing varies in different individuals; in some, being from seventy to one hundred cubic inches, sometimes over, and sometimes under this amount. In diseases of the lungs, the amount varies very much. Right in these air cells or vesicles, takes place, intermixture of gases and asmatic passage in and out of the circulation of vapor and gases; within these vesicles no important chemical change takes place, though changes of great vital import.

The pulmonary capillary vessels everywhere surround these air vesicles, hence, are much more numerous than the vesicles themselves. As the chest rises and falls, there is necessarily a displacement of atmosphere in both cases that just equals each other, hence there is no lifting either way in this apparent displacement: otherwise, fatal collapses might result.

The bulk of food and air taken into the body in twelve months, is given out again in just about equal bulk, there being no essential difference, so that what is taken into the body and given out again, has undergone important chemical changes without any material change in volume. This chemical change has been all important to the individual *vis viva*, having been developed just equal to the amount of dynamical disturbance needed; a perfect system of correlation or conservation forces having existed when the balances were unbroken, which means physiology, and when broken, means pathology. The sum of life, then, consists of a system of chemical and physical forces, always vibrating in harmony with themselves and the outer world, being at the same time part and parcel of the universe.

Reproduction comes under the head of normal physiology, and like crystals, prototypal forms are reproduced, as when any crystalline salt is dissolved and re-crystalized, the same type as the original is reproduced under like circumstances; these are called typal forms.

Prof. Agassiz has been lecturing on eggs, and he asserts that " all living beings, whatever their diversity of form, have grown up from eggs, which are at first all precisely similar, deviations take place, little understood, that eventually change these beings into widely different animals." The editor remarks, " this explains where every thing comes from, except eggs."

In conclusion. The main object in taking food and air into the system is for the purpose of keeping up a supply of force, which is being constantly expended by vital and chemical changes going on, and when food is withheld, or rendered unavailable, as during sickness, chemical changes still go on in the body at the expense of vital force ; hence, debility, requiring suspension of voluntary motion in proportion to vital exhaustion.

When persons die from starvation, they die mainly from *inanition*, and syncope ends the career, similar to that of hemorrhage when air is withheld, apnœa or asphyxia follows. Longevity or old age may be regarded as a loss of balance in the forces, the chemical gradually gaining a predominance over vital or nutrition, and the individual really becoming a fossil, so to speak, death being the grand climax in life's drama. Food and air enter the body as living factors, carrying in potential energy by oxidation, which becomes dynamical force, or *vis viva*, then leaving the body as dead matter, *so-called*, having done work, and died in giving life to the body.

---

